# Patient safety culture in Peking University Cancer Hospital in China: baseline assessment and comparative analysis for quality improvement

**DOI:** 10.1186/s12913-019-4837-z

**Published:** 2019-12-28

**Authors:** Xiyao Zhong, Yuqin Song, Christine Dennis, Donna J. Slovensky, Lim Yee Wei, Jie Chen, Jiafu Ji

**Affiliations:** 1 Key Laboratory of Carcinogenesis and Translational Research (Ministry of Education/Beijing), Peking University Cancer Hospital and Institute, Beijing, 100142 China; 2 Australian Council Healthcare Standards, Ultimo, Australia; 3 Professor and Senior Associate Dean for Academic and Faculty Affairs, School of Health Professions, University of Alabama at Birmingham, Birmingham, USA; 4 Department of Medicine, Yong Loo Lin School of Medicine, National University of Singapore, Singapore, Singapore; 5 Department of Health Services Administration, School of Public Health, University of Maryland, College Park, MD USA

**Keywords:** Patient safety, Hospital survey on patient safety culture (HSOPSC), Chinese hospitals

## Abstract

**Background:**

Limited information is available regarding the patient safety culture in Chinese hospitals. This study aims to assess the patient safety culture in Peking University Cancer Hospital and to identify opportunities for improving the organization’s safety culture.

**Methods:**

A cross-sectional study was conducted in April 2018 and 2019, respectively. Data on patient safety culture were collected from clinical and administrative staffs using the Hospital Survey on Patient Safety Culture (HSOPSC).

**Results:**

Twelve composite dimension variables were hierarchically clustered. Three highest positive response dimensions include ‘Organizational Learning and continuous improvement’ (92.9%), ‘Teamwork within units’ (89.7%), and ‘Hospital management support for patient safety’ (83.7%), while 3 lowest positive response dimensions included ‘Frequency of events reported’ (43.9%), ‘Non-punitive response to error’ (51.1%), ‘Communication openness’ (52.2%), and ‘Staffing’ (53.7%). Compared to the average scores of the United States, the scores of the Peking University Cancer Hospital was significantly lower on ‘Communication openness’ and ‘Frequency of events reported’. After targeted continuous improvement based on results in 2018, all 12 dimensions surprisingly increased in the safety culture conducted in 2019.

**Conclusion:**

Inadequate feedback and communications about error and lack of communication openness are key challenges for patient safety in the delivery of care in this hospital. Results of this baseline survey indicate the need for a modified approach and attention to context when designing interventions aimed at improving the safety culture in this organization.

## Background

Patient safety is receiving growing attention in China. Developing and maintaining a positive patient safety culture among clinical and administrative staff is widely recognized as a crucial element in the improvement of patient safety in healthcare organizations. A culture of safety has been defined as “a collaborative environment in which skilled clinicians treat each other with respect, leaders drive effective teamwork and promote psychological safety, teams learn from errors and near misses, caregivers are aware of the inherent limitations of human performance in complex systems (stress recognition), and there is a visible process of learning and driving improvement through debriefings” [1]. Simply put, safety culture refers to the beliefs, values, perceptions and attitudes of patient safety shared among members of the organization. It is recognized as a key element in improving quality of care, and in decreasing/preventing medical errors. Assessment of the safety culture helps leaders in healthcare organizations to determine areas for patient safety improvement, to evaluate the success of patient safety interventions, to benchmark against other organizations, and to meet regulatory requirements.

The Agency for Healthcare Research and Quality (AHRQ) is a unit within the U.S. Department of Health and Human Services dedicated to “producing evidence to make health care safer, higher quality, more accessible, equitable, and affordable” [2]. In support of this mission, AHRQ developed a tool to assess healthcare organizational culture regarding patient safety. The Hospital Survey on Patient Safety Culture (HSOPSC) was released in 2004 and has been widely used by healthcare organizations in the U.S. and internationally [3]. The HSOPSC measures an organization’s safety culture based on 42 items that measure 12 composites (10 safety dimensions and 2 outcome dimensions), staff perceptions on patient safety issues, and adverse events reporting. The English language version of the survey is available from the AHRQ.gov website [2].

In this study, a Chinese-translated version of the HSOPSC tool was used to conduct a baseline assessment and comparative analysis of the patient safety culture in Peking University Cancer Hospital (PUCH) to identify opportunities for improvement in the organization’s safety culture.

The Peking University Cancer Hospital, one of the most comprehensive cancer centers in China, has a total capacity of 790 beds with all major medical specialties and services represented. The hospital, located in the capital city of Beijing, is an academic medical center with a strong basic and clinical research focus in addition to providing cancer treatments using a wide variety of interventional approaches.

## Methods

### Participants and assessment tool

The HSOPSC provides a comprehensive assessment of patient safety culture. The guideline is comprised of 42 items that measure 12 composites (10 safety dimensions and 2 outcome dimensions).

The investigation instrument was a validated Chinese version of the HSOPSC [4]. It has also been used in many Chinese studies [5, 6]. The translated HSOPSC was delivered as an anonymous online survey, during April 2018 and 2019, respectively, to all the clinical and non-clinical hospital staff members whose jobs contributed to patient safety. Specifically, these respondents, included physicians, nurses, clinical and non-clinical staff, pharmacy and laboratory staff, dietary and radiology staff, supervisors, and hospital managers. The defined study sample comprised 1931 individuals.

### Statistical analysis

Data were analyzed using SPSS 20.0. Univariate analysis was conducted to summarize the demographic characteristics of respondents. Respondents’ gender, job category, work unit, experience (in current hospital, department, and work area), and weekly hours of work were presented.

The HSOPSC is comprised of 42 items that measure 12 composites (i.e. each composite was calculated based on responses to 3–4 items). Items were scored on a five-point frequency scale and included both positively and negatively worded items. For each positively worded item, the percentage of positive responses was calculated, i.e., the percentage of respondents answering the question as “Strongly Agree/Agree” or “Always/Most of the time”.

Similarly, for reverse worded items, disagreement indicates a positive response, so the responses ‘Strongly Disagree/Disagree’ or ‘Never/Rarely’ are considered positive.

Composite level scores were computed by summing the items within the composite scales and dividing by the number of items with non-missing values. Cronbach’s Alpha was used to test for the internal consistency and reliability of the 12 composites.

The Pearson’s correlation coefficient was used to examine the association between frequency of events reported and overall perception of safety and the remaining 10 composites at the bivariate level. The ‘number of events reported’ question asks for the number of adverse events reports the individual had submitted in the previous 12 months.

We used generalised estimating equations with an independence working correlation to fit a proportional odds logistic regression model for number of events and patient safety grade. The two outcomes were grouped into 3 categories: ‘Poor of Failing’, ‘Acceptable’, and ‘Excellent/Good’ for patient safety, and ‘> 5’, ‘1–5’, and ‘no events’ for number of events.

Finally, the percent positive responses for each composite variable calculated for Peking University Cancer Hospital, and published data from the Kingdom of Saudi Arabia, Lebanon, Turkey and the United States were compared collectively. A one-sample t-tests was used to compare the results of Peking University Cancer Hospital against these other countries in pairs.

## Results

### Respondents’ characteristics

A total of 1562 of the 1931 questionnaires was returned for a response rate of 80.9%. Respondents’ characteristics are presented in Table 1. The majority of respondents were female (72.7%). Almost one-third of respondents worked in surgical units (29.3%), while 20.5% worked in medical units, 27.3% in diagnostic units, 14.3% in administration, and 8.7% in other units. Doctors comprised 22.1% of the sampled respondents, 38.8% were nurses, 25.5% were technicians, and 13.6% were administrative staff. A third of respondents had between 1 and 5 years of experience (32.5%), while 23.1% had between 6 and 10 years of experience. Almost three-quarters of respondents indicated that their work required direct contact with patients (72.7%).

**Table 1:** Characteristics of respondents of the Hospital Survey on Patient Safety Culture (HSOPSC) conducted at the Peking University Cancer Hospital

Characteristics	N	%
Gender		
Male	426	27.3
Female	1136	72.7
Job category		
Doctor	345	22.1
Nurse	606	38.8
Technician	398	25.5
Administration	213	13.6
Work unit		
Administration	223	14.3
Diagnostics	426	27.3
ICU, operating room, anesthesiology	136	8.7
Medical	320	20.5
Surgical	457	29.3
Clinical department or not		
Yes	994	63.6
No	568	36.4
Experience in current hospital (years)		
Less than 1	94	6.0
1 to 5	507	32.5
6 to 10	361	23.1
11 to 15	248	15.9
16 to 20	153	9.8
21 years or more	199	12.7
Experience in current department (years)		
Less than 1	139	8.9
1 to 5	585	37.5
6 to 10	400	25.6
11 to 15	235	15
16 to 20	111	7.1
21 years or more	92	5.9
Experience in current work area (years)		
Less than 1	53	3.4
1 to 5	499	31.9
6 to 10	409	26.2
11 to 15	254	16.3
16 to 20	170	10.9
21 years or more	177	11.3
Hours of work per week		
< 20 h	8	0.5
20–39 h	202	12.9
40–59 h	1120	71.7
60–79 h	182	11.7
80–99 h	29	1.9
100 h	21	1.3
Job involves direct contact with patients		
Yes	1135	72.7
No	427	27.3
Patient safety grade		
Excellent	540	34.6
Good	761	48.7
Acceptable	231	14.8
Poor	25	1.6
Failing	5	0.3
Number of adverse events reported		
No events	945	60.5
1 to 2 event reports	453	29.0
3 to 5 event reports	104	6.7
6 to 10 event reports	34	2.2
11 to 20 event reports	16	1.0
21 event reports or more	10	0.6

Approximately half of respondents gave the hospital a ‘Good’ patient safety grade (48.7%). 60.5% of the sampled respondents reported no adverse safety events, approximately a third (29%) reported 1 to 2 events, and 6.7% reported 3 to 5 events. It is notable that only 1.6% of respondents reported 11 or more events although this still equates to several hundred reported events.

### Patient safety culture composite scores

The twelve composite variable scores were hierarchically clustered. Cluster I (highest positive response) grouped ‘Learning and continuous improvement’ (92.9%), ‘Teamwork within units’ (89.7%), and ‘Hospital management support for patient safety’ (83.7%). Cluster II (lowest positive response) included ‘Frequency of events reported’ (43.9%), ‘Non-punitive response to error’ (51.1%), ‘Communication openness’ (52.2%), and ‘Staffing’ (53.7%).

Results reported in Table 2 indicate that internal consistency and reliability of the 12 composites were acceptable, with Cronbach’s values ranging from a low of 0.53 (Staffing) to a high of 0.87 (Teamwork within units). According to the HSOPSC user’s guide [7], a Cronbach’s α 0.6 is acceptable, whereas Bowling [8] states that a value of 0.5 or above indicates good internal consistency. However, when using psychological constructs, lower values of Cronbach’s α are expected due to the diversity of the constructs being measured [9].

**Table 2:** Distribution of positive responses and scores for survey composites and items

Composites and survey items	Average positive response (%)^*^	Mean	SD
Overall perception of safety (Cronbach’s a = 0.61)	74.6	4.0	0.7
Patient safety is never sacrificed to get more work done	85.5	4.2	1.0
Our policies and procedures and systems are effective in preventing errors	77.4	4.0	0.9
It is just by chance that more serious mistakes do not happen around here(R)**	71.4	4.0	1.0
We have patient safety problems in this unit(R)	64.0	3.8	1.0
Supervisor/Manager expectations & actions promoting patient safety (Cronbach’s a = 0.78)	81.6	4.1	0.6
My supervisor/manager says a good word when he/she sees a job done according to established patient safety procedures	79.2	4.0	0.8
My supervisor/manager seriously considers staff suggestions for improving patient safety	89.1	4.3	0.7
Whenever pressure builds up, my supervisor/manager wants us to work faster, even if it means taking shortcuts(R)	72.3	3.9	1.0
My supervisor/manager overlooks patient safety problems that happen over and over(R)	85.7	4.2	0.8
Organizational learning and continuous improvement (Cronbach’s a = 0.79)	92.9	4.4	0.6
We are actively doing things to improve patient safety	96.4	4.5	0.6
Mistakes have led to positive changes here	95.1	4.4	0.7
After we make changes to improve patient safety, we evaluate their effectiveness	87.3	4.2	0.7
Teamwork within units (Cronbach’s a = 0.87)	89.7	4.3	0.7
Staff supports one another in this unit	92.8	4.4	0.8
When a lot of work needs to be done quickly, we work together as a team to get the work done	92.8	4.4	0.7
In this unit, people treat each other with respect	91.6	4.4	0.8
When members of this unit get really busy, other members of the same unit help out	81.6	4.1	0.9
Staffing (Cronbach’s a = 0.53)	53.7	3.5	0.7
We have enough staff to handle the workload	75.9	4.0	1.0
Staff in this unit work longer hours than is best for patient care (R)	39.7	3.1	1.2
We use more agency/temporary staff than is best for patient care (R)	65.9	3.8	1.0
When the work is in “crisis mode” we try to do too much, too quickly (R)	33.4	3.0	1.2
Hospital management support for patient safety (Cronbach’s a = 0.74)	83.7	4.2	0.7
Hospital management provides a work climate that promotes patient safety	80.8	4.1	0.8
The actions of hospital management show that patient safety is a top priority	90.7	4.3	0.7
Hospital management seems interested in patient safety only after an adverse event happens (R)	75.9	4.0	0.9
Hospital handoffs & transitions (Cronbach’s a = 0.86)	73.1	4.0	0.7
Things “fall between the cracks”, i.e., things might go uncontrolled and get lost when transferring patients from one unit to another (R)	55.1	3.6	0.9
Important patient care information is often lost during shift changes (R)	87.2	4.3	0.8
Problems often occur in the exchange of information across hospital units (R)	75.4	4.0	0.8
Shift changes are problematic for patients in this hospital (R)	74.6	4.0	0.9
Communication openness (Cronbach’s a = 0.57)	52.2	3.5	0.7
Staff will freely speak up if they see something that may negatively affect patient care	70.0	3.9	0.9
Staff feel free to question the decisions or actions of those with more authority	21.3	2.9	1.0
Staff are afraid to ask questions when something does not feel right (R)	65.3	3.8	1.0
Feedback and communications about error (Cronbach’s a = 0.76)	77.6	4.1	0.7
We are given feedback about changes put into place based on event reports	76.8	4.1	0.8
We are informed about errors that happen in this unit	73.9	4.1	0.9
In this unit, we discuss ways to prevent errors from happening again	82.1	4.2	0.8
Frequency of events reported (Cronbach’s a = 0.89)	43.9	3.3	1.0
When a mistake is made, but is caught and corrected affecting the patient, how often is this reported?	44.0	3.4	1.1
When a mistake is made, but has no potential to harm the patient, how often is this reported?	41.5	3.3	1.1
When a mistake is made that could harm the patient, but does not, how often is this reported?	46.2	3.4	1.2
Non-punitive response to error (Cronbach’s a = 0.68)	51.1	3.4	0.8
Staff feel like their mistakes are held against them (R)	45.7	3.2	1.1
When an event is reported, it feels like the person is being written up, not the problem (R)	75.0	3.9	1.0
Staff worry that mistakes they make are kept in their personnel file (R)	35.5	3.0	1.1
Teamwork across hospital units (Cronbach’s a = 0.84)	76.2	4.0	0.7
Hospital units do not coordinate well with each other and this might affect patient care (R)	65.7	3.8	1.0
There is good cooperation among hospital units that need to work together	79.1	4.0	0.8
It is often not easy to work with staff from other hospital units (R)	75.0	3.9	0.9
Hospital units work well together to provide the best care for patients	85.2	4.2	0.8

*The composite-level percentage of positive responses was calculated using the following formula: (number of positive responses to the items in the composite/total number of responses compared with the items (positive, neutral, and negative) in the composite (excluding missing responses))*100

**(R) Negatively worded items that were reverse coded

### Correlations between patient safety culture composites

Table 3 shows correlations between the 12 patient safety culture composites, which were found to be significantly correlated. Within the composite on ‘Frequency of events reported’, the strongest correlation was observed for ‘Feedback and communication about error’ (Pearson’s r = 0.41), while the weakest correlation was for that on ‘Staffing’ (Pearson’s r = 0.17) and ‘Non-punitive response to error’ (Pearson’s r = 0.17).

**Table 3:** Correlations between patient safety culture composites^*^

	Frequency of events reported		Overall perception of safety	
	Pearson’s r	P	Pearson’s r	P
Supervisor/Manager expectations and actions promoting safety	0.27	< 0.001	0.63	< 0.001
Organizational learning-continuous improvement	0.26	< 0.001	0.61	< 0.001
Teamwork within hospital units	0.22	< 0.001	0.54	< 0.001
Staffing	0.17	< 0.001	0.49	< 0.001
Hospital management support for patient safety	0.26	< 0.001	0.58	< 0.001
Hospital handoffs and transitions	0.27	< 0.001	0.57	< 0.001
Communication openness	0.33	< 0.001	0.47	< 0.001
Feedback and communication about errors	0.41	< 0.001	0.49	< 0.001
Non-punitive response to error	0.17	< 0.001	0.50	< 0.001
Teamwork across hospital units	0.24	< 0.001	0.53	< 0.001

^*^N = 1562, correlation is significant at the 0.01 level (2-tailed)

As for the composite on overall perception of patient safety, the strongest correlation was for ‘Management expectations and actions promoting safety’ (Pearson’s r = 0.63), and the weakest was for ‘Communication openness’ (Pearson’s r = 0.47).

### Generalized estimating equations for the patient safety composite scores and respondent characteristics against the patient safety grade and the number of events reported

As shown in Table 4, five safety composites were found to be significantly associated with patient safety grade. Patient safety grades has 2.3 higher odds (95% CI: 1.5, 3.4) for every unit increase in ‘Hospital handoffs & transitions’, 2.1 (95% CI: 1.3, 3.2), for every unit increase in ‘Organizational learning-continuous improvement’, 2.0 (95% CI: 1.3, 3.1), for every unit increase in ‘Hospital management support for patient safety’, 1.6 (95% CI: 1.1, 2.4), for every unit increase in ‘Supervisor/Manager expectations’ & ‘Actions promoting patient safety’, 1.4 (95% CI: 1.0, 2.0), and for every unit increase in ‘Communication openness’.

**Table 4:** Results of the generalized estimating equations for the patient safety composite scores and respondent characteristics

	Patient safety grade		Number of events reported	
	OR (95%CI)	P	OR (95%CI)	P
Patient safety culture composites				
Supervisor/Manager expectations & actions promoting patient Safety	1.6 (1.1,2.4)	0.02	1.3 (1.0,1.7)	0.07
Organizational learning-continuous improvement	2.1 (1.3,3.2)	0.001	0.9 (0.7,1.2)	0.51
Teamwork within units	1.2 (0.9, 1.7)	0.22	0.9 (0.7,1.1)	0.34
Communication openness	1.4 (1.0,2.0)	0.03	0.9(0.8,1.1)	0.50
Feedback and communications about error	1.3(0.9,1.8)	0.09	1.0(0.8,1.3)	0.95
Non-punitive response to error	1.1(0.8,1.4)	0.34	1.1(0.9,1.3)	0.23
Staffing	1.2 (0.9,1. 6)	0.27	0.8 (0.7,0.9)	0.044
Hospital management support for patient safety	2.0 (1.3,3.1)	0.001	1.4 (1.0,1.8)	0.024
Hospital handoffs & transitions	2.3 (1.5,3.4)	< 0.001	0.7 (0.5,0.9)	0.006
Teamwork across hospital units	0.8 (0.5,1.3)	0.42	0.9 (0.7,1.2)	0.37
Gender				
Male	1.1 (0.8,1.7)	0.55	1.3 (1.0,1.7)	0.088
Female	1		1	
Job category				
Doctor	1.8(0.6,5.2)	0.29	3.1(1.2,8.1)	0.019
Nurse	1.0(0.3,2.8)	0.96	3.1(1.2,7.9)	0.020
Technician	2.1 (0.8,5.8)	0.16	1.5 (0.6,3.8)	0.42
Administrator	1		1	
Work unit				
Medical	1.1 (0.6,2.1)	0.77	1.2 (0.8,1.8)	0.45
Surgical	1.4 (0.8,2.5)	0.26	0.8 (0.5,1.1)	0.18
ICU, operating room, anesthesiology	1.9(0.9,4.1)	0.11	0.4(0.3,0.7)	0.002
Administration	2.2(0.8,6.0)	0.13	0.8(0.3,2.0)	0.64
Diagnostic	1		1	
Clinical department or not				
Yes	0.8 (0.5,1.3)	0.33	0.9 (0.6,1.4)	0.76
No	1		1	
Experience in current hospital (years)				
Less than 1	0.6 (0.2,1.5)	0.26	0.4 (0.2,0.7)	0.002
1 to 5	0.6 (0.3,1.1)	0.11	0.9 (0.6,1.3)	0.55
6 to 10	0.5 (0.3,0.9)	0.022	0.9 (0.6,1.3)	0.51
11 to 15	0.4 (0.2,0.8)	0.008	0.9 (0.6,1.4)	0.67
16 to 20	0.6 (0.3,1.2)	0.12	0.7 (0.5,1.2)	0.21
21 years or more	1		1	
Job involves direct contact with patients				
Yes	0.8 (0.5,1.2)	0.26	1.3 (1.0,1.9)	0.055
No	1		1	

An increase in ‘Hospital handoffs & transitions’, ‘Hospital management support for patient safety’, and ‘Staffing’ led to higher odds of reporting a higher number of events.

OLS was tested as sensitivity analyses. Findings were similar.

### Comparison of the 12 composite means with international and regional findings

**Fig. 1:**
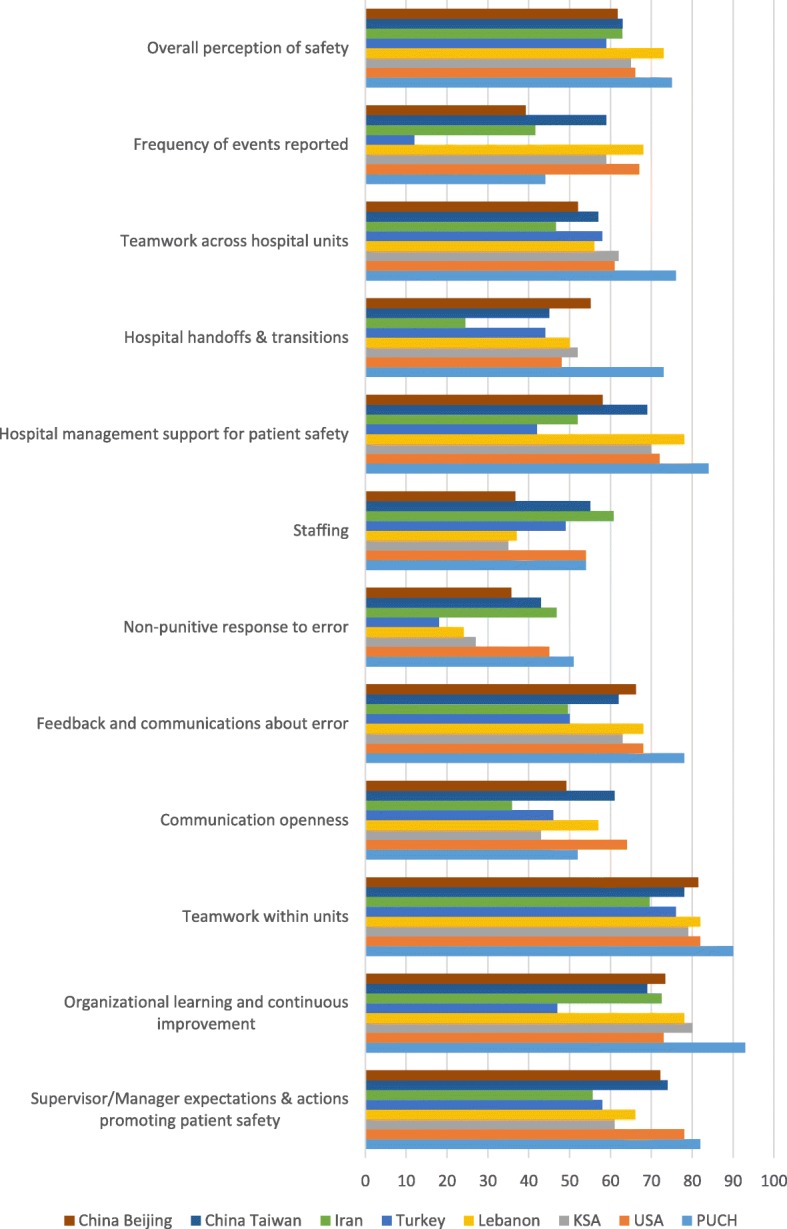
Composite-level average per cent positive response for PUCH compared to that of other countries and regions

Data in Fig. 1 show the variation in differences of patient safety culture composite means in the United States [10], Beijing [11], and other countries or regions [12–16]. Taking into account the differences between health policy and economic conditions, we focus our discussion on another survey in Beijing [11] similar to our hospital’s overall situation. Compared to average scores of hospitals in Beijing, the scores of PUCH were significantly higher in all 12 composites. Since China is a developing country, we also concerned about the differences with developed countries such as the United States [10]. Compared to the United States, the sampled hospital scores were significantly lower on ‘Communication openness’ and ‘Frequency of events reported’, and other scores were significantly higher except ‘Staffing’.

### Comparison of the 12 composites data of 2018 with the data of 2019

The HSOPSC was conducted in April 2019, with a total of 1562 of the 1972 questionnaires was returned for a response rate of 79.2%. Surprisingly, all 12 dimensions increased in the safety culture conducted in 2019 (Table 5), and 4 dimensions remain in the lowest positive response group, include ‘Frequency of events reported’ (47.9, 43.9% in 2018), ‘Non-punitive response to error’ (54.3, 51.1% in 2018), ‘Communication openness’ (55.3, 52.2% in 2018), and ‘Staffing’ (55.9, 53.7% in 2018). It indicates that results of the baseline survey in 2018 might be helpful in designing interventions aimed at improving the safety culture in this organization.

**Table 5:** Distribution of positive responses and scores for survey composites and items in 2019 compared with 2018

Composites and survey items	Average positive response of 2019 (%)	Average positive response of 2018 (%)	US Average (%)
Overall perception of safety	78.6	74.6	66
Supervisor/Manager expectations & actions promoting patient safety	84.0	81.6	78
Organizational learning and continuous improvement	94.2	92.9	73
Teamwork within units	90.3	89.7	82
Non-punitive response to error	54.3	51.1	45
Staffing	55.9	53.7	54
Hospital management support for patient safety	86.3	83.7	72
Teamwork across hospital units	77.9	76.2	61
Hospital handoffs & transitions	76.7	73.1	48
Communication openness	55.3	52.2	64
Feedback and communications about error	79.9	77.6	68
Frequency of events reported	47.9	43.9	67

## Discussion

‘Frequency of events reported’ (43.9%), ‘Non-punitive response to error’ (51.1%), ‘Communication openness’ (52.2%), and ‘Staffing’ (53.7%) had the lowest scores. Of the two outcome composites, ‘Frequency of events reported’ and ‘Overall perception of patient safety’, the strongest correlation was for ‘Feedback and communication about error’ (Pearson’s r = 0.41) and ‘Management expectations and actions promoting safety’ (Pearson’s r = 0.63). Five safety composites were found to be significantly associated with patient safety grade.

### Comparison of the 12 composites means with international and regional findings

Compared to Beijing, the sampled hospital scores were significantly higher in all 12 composites, suggesting the sampled hospital has a relatively positive safety culture compared with the average safety culture level in hospitals in Beijing. Compared to the United States, the sampled hospital scores were significantly lower on ‘Communication openness’ and ‘Frequency of events reported’, which is consistent with the hierarchy of clustered findings. PUCH performance is weak in the dimensions ‘communication openness’ and ‘frequency of events reported’, indicating a need for management changes to improve performance. There is no significant difference in ‘Staffing’ between the sampled hospital and the United States average, which is clustered in the lowest group.

### Policy implications for hospital management

A culture of safety is fundamental when seeking improvement of quality in healthcare delivery. It is defined as shared values, attitudes and perceptions of safety within an organization that have the goal of minimizing risk of patient harm. It includes the following components: (1) recognizing that high-risk settings are more error prone, (2) nurturing a blame-free environment, (3) management allocating resources for safety concerns, and (4) collaboration among professional disciplines to seek solutions. Organizations with a positive safety culture are characterized by mutual trust, open communication, shared perceptions about safety issues, and confidence about the effectiveness of preventive measures [3].

The findings of this study suggest that hospital leadership must be concerned that interventions focus on feedback and communication about error, as well as communication openness. As stated by Sammer et al. [17], “a common theme running through the literature suggests the role of senior leadership is a key element to designing, fostering, and nurturing a culture of safety.” Building safety cultures and improving the quality and safety of care will not occur in environments where staff do not feel supported to communicate and report errors or near miss incidents.

Repeated measurement over several years is needed to track performance evolution in these dimensions. Greater attention to performance in the individual dimensions must be paid by hospital management in order to evaluate organizational readiness to deploy patient safety strategies. In the future, the hospital management should adjust the patient safety strategy based on the results of these two surveys to improve the management and consciousness level to construct a good safety culture in the hospital.

## Limitations

Our study has some limitations. We only studied the safety culture of our own hospital. We are an oncology hospital and the conclusions of our study may not be applicable to other tertiary hospitals. However, our study may be helpful for hospitals willing to assess and improve their patient safety culture in China.

## Conclusion

Patient safety is considered to be crucial for healthcare organizations that want to improve overall performance and quality of services. Assessment of patient safety culture in these healthcare organizations is necessary, and more importantly, make changes based on the results of such assessments. As for our surveys, this hospital has potential for improvement in feedback and communications about error and communication openness. A modified approach and attention are needed to context when designing interventions aimed at improving the safety culture in this organization.

## Data Availability

The datasets used and/or analysed during the current study are available from the corresponding author on reasonable request.

## Abbreviations

AHRQ: Agency for Healthcare Research and Quality
HSOPSC: Hospital Survey on Patient Safety Culture
PUCH: Peking University Cancer Hospital
